# Hospital and Institutional News

**Published:** 1921-03-19

**Authors:** 


### HOSPITAL AND INSTITUTIONAL NEWS.

### NATIONAL RELIEF FUND CLOSED.

We are informed that the whole of the balance of the National Relief Fund, originally called the Prince of Wales's Fund, has now been distributed. The most important grants since the issue of the last report include £700,000 to meet war deficits of voluntary hospitals in the United Kingdom, £500,000 to the Central Committee on Women's Employment for training displaced women war workers, and £50,000 to the War Seal Foundation for paralysed ex-Service men. From the commencement of the Fund until September 1918 the Prince of Wales was Treasurer and defrayed all clerical expenses from his private purse; since that date the affairs have been entirely managed, by the Prince's desire, by the Executive Committee.

### SHOULD MATRONS HAVE NURSING COMMITTEES?

In view of previous events, the annual meeting of Cumberland Infirmary could hardly, perhaps, have been expected to pass without some reference to them. Dr. Barnes, the President, was in the chair, and after his address, which was full of good points, and Mr. E. W. Stead, the treasurer, had reported a financial deficit of nearly £10,000, Dr. Lediard mentioned certain "omissions from the report." He did not propose to dwell on these, but because there has been trouble at the Infirmary they want, he said, a change of committee-men, and they must have it. To raise money for the Infirmary, money is wanted from the works, which will not subscribe unless represented upon the Committee. He hopes that these bodies, therefore, will be represented upon the Committee. New blood is wanted, but he trusts that the old clouds will pass under the new President's leadership, for their President is acquainted practically with the work of the wards, with the sick, and with the domestic and general administration. Newcomers will already recognise a different atmosphere, of hope and confidence and willingness, from which much is hoped under the new matron. But he wished to utter a word of warning. The report declared that a nursing committee has been appointed. Last year they did without such a committee, and an experienced and capable matron should not be harassed by such a body. The report was passed, and, now that Dr. Lediard has publicly made his points, we hope that the new year will be one of quiet progress. But the principles of workmen's representation and of a nursing committee are important. On the former there can be no two opinions, but the advisability of a nursing committee is one that can be legitimately debated.

### NEW SECRETARIAL APPOINTMENT.

We congratulate Mr. G. N. Pitt on his appointment as secretary of the London Jewish Hospital. Mr. Pitt has been for about two years steward of St. John's College, Oxford, and was previously for many years steward of St. Mary's Hospital, Paddington. For a long period he was one of the honorary secretaries of the Incorporated Association of Hospital Officers, and he is well known and very popular in hospital circles.

### DANGEROUS DRUGS DAY BY DAY.

Questions continue to be addressed to the Home Secretary with regard to the draft Regulations of the Dangerous Drugs Act. Mr. Shortt informed Major Bennett that in view of representations from the British Medical Association certain modifications have been made as to the records required to be kept by medical practitioners and as to the markings of packages containing drugs, the latter in view of the fact that it is sometimes undesirable that the patient should obtain information as to the contents. The Secretary of State for India informed an inquirer that it is not proposed to introduce legislation in trade similar to the Act, as the regulation of the traffic in and use of such drugs is already strictly controlled under a variety of Local and Imperial Acts. The Home Secretary later announced that he had decided to appoint a small committee, to report to him on cases in which an agreement with interested parties as to the Regulations had not been come to. We understand that some special regulations to apply to hospital dispensaries are to be formulated.

### INACTION AT GRAHAMSTOWN.

Conditions at the Albany General Hospital, Grahamstown, Cape Province, have been made the subject of an inquiry by the Government, following charges against the administration made by the parents of a patient who died in the hospital of typhoid or paratyphoid. The report of the Commissioners of Inquiry points out that in numerous cases certain improvements which have been suggested by the health officers since 1904 as urgently necessary have not been carried out, and criticises successive hospital boards for not having made earlier efforts to rebuild the institution. It comments adversely upon the proximity of the infectious disease building to the general hospital, the existing sanitary arrangements, and on the small proportion of trained nurses to untrained nurses, and it impresses upon the board the necessity for probationers being instructed in the elements of hygiene before undertaking nursing responsibilities. It further urges that natives should be entirely separated from the European wards. There seems need for radical action in connection with the affairs of this hospital, and we hope the residents of Grahamstown will see to it that such action is not longer delayed. We understand that the matron has intimated to the Committee her intention to resign; that the hospital is urgently in need of funds; and that tradesmen's accounts have been four and six months overdue. Albany Hospital is one where, according to the latest available report, patients are received who pay from £1 15s. to £3 10s. a week. There are eighty beds, with an average number occupied of sixty-three, and the nursing staff consists of a matron, four sisters, and fourteen probationers. The Commissioners pay a tribute to the discipline maintained among the staff, which they state is doing its best under difficult conditions.

### THE MAIDSTONE PLAN.

Mr. Alfred Marshall, formerly of Winchester, but now a builder at Maidstone, Kent, has sought to interest Hampshire people by sending to them an account of the efforts made in Maidstone on behalf of the West Kent Hospital there. He describes the appeal made first to the women of Maidstone, to prevent some wards from being closed, pointing out that the plan was proposed by the workers themselves in the building and other trades. A contribution according to the donor's wages was suggested, and Mr. Marshall mentions one motor-works where from 8d. to 1s. a week is subscribed; another firm of bun-flour makers, where the rate is from 2d. to 8d.; another where the men contribute according to their wages, and the girls 2d. The number of subscribers in these three firms alone amounts, he tells us, to over 1,300; the number of the second firm's subscribers is not given. In the building (his own) trade every man, he says, gives 8d. a week. The contributions continue for twelve months, or until a subscriber has completed one week's wages, provided that the hospital committee shall receive £100,000. The plan, he adds, admits a wife and children, as well as the head of the family, to the hospital. Mr. Marshall invites the attention of Winchester workers to the Kent plan. He has done good service in this exchange of ideas, which we wish were made more often. The Kent plan is worthy of study everywhere.

### £10,000 FOR ST. BARTHOLOMEW'S.

The large donation of £10,000 announced at the recent visit of the Queen to St. Bartholomew's is, we learn, the outcome of the generosity of Alderman Sir John Bell, Sheriff of the City in 1902 and Lord Mayor in 1907-8.

### UNEMPLOYMENT AND HOSPITAL BUILDING.

Because one good turn deserves another the Newport Workmen's Hospital Fund, when handing over its handsome contribution of £9,000 for the year 1920 to the general funds of the Royal Gwent Hospital (an increase of £2,000 on the previous year's total), makes to the directors of the institution a certain request. In consequence of the large amount of unemployment in the town and county, the fund invites the committee to put in hand immediately such work upon the hospital extension scheme as will help to ease some of the present distress. The request is one which the directors are certain to deal with most sympathetically, but the present times are not, of course, the best for extensions to be carried out. Will the building trades and contractors *also* help to make such action possible? If co-operation is needed, of which there can be no doubt, it must be given all round; and it is remarkable that the request mentioned has come from a body which is one of those concerned with relieving distress, and not, also, from the contractors and trades unions concerned in building.

### AN EXPENSIVE SCHEME STOPPED.

Lambeth Borough Council some months ago decided to build a tuberculosis dispensary on a piece of vacant land adjoining their Town Hall at Brixton, and forwarded their plans with full details to the Minister of Health for his approval. The Minister has now notified the Council that the estimated cost of the new building is too great to justify the sanction of the scheme; but if the Council desire, their Borough Engineer and Medical Officer of Health are invited to confer with the Medical and Architectural Departments of the Ministry with a view to providing the necessary accommodation at less cost. The Council have agreed to this course, and probably a conference may result in both parties being satisfied with a more modified scheme. At present the tuberculosis dispensary is in a house some distance away from the Town Hall, and, although connected by telephone, there cannot be that co-ordination between the officers that there should be to ensure the easy working of the dispensary and the Public Health Department of the Council.

### INCURABLES AND LONGEVITY.

Some patients at the Royal Hospital and Home for Incurables, Putney, have sojourned there for an astonishing number of years. The death of Miss Sarah Elizabeth Cruttwell at the advanced age of ninety-three shows with what care the patients are tended. For fifty-six years she had been a pensioner of the institution. From 1865 to 1885 she was drawing £20 a year whilst her relatives were alive, but when at last she was left alone she was taken into the home, where she remained for thirty-six years till her death. Although thirty-six years is a long time to remain in such an institution, there are other inmates who have been there longer. For thirty-seven years Miss Tillee has been bedridden there; Miss Curtis and Mr. Harry Hawkes, whose illness is a tragedy of the Indian Mutiny, have both been in the home for fifty-one years. In addition to 233 incurable patients in the Putney home, there are 700 pensioners drawing £20 a year to help in their maintenance at home.

### THE COST OF WATER AND TELEPHONES.

The Secretary of the British Hospitals Association has received a letter from the Metropolitan Water Board stating that hospitals were duly considered in the provisions of the new Metropolitan Water Board (Charges) Bill, and where such institutions are wholly or partly supported by endowments or voluntary constitutions, and not carried on for purposes of private gain or profit, although the basis of the rate shall be by measure, a discount up to thirty-five per cent. may be granted by the Board in respect of the accounts. We think we are right in saying that when the Board absorbed the private water-supply companies it was Lord Knutsford who was successful in obtaining a concession for hospitals, arranging for payment according to rateable value instead of by meter, and although the Board reserved the right to adopt the second method at their discretion, the hospitals have of recent years paid on assessment and benefited greatly thereby. An inquiry addressed to the Postmaster-General by the British Hospitals Association has resulted in no preferential treatment, as the authority, unlike the Water Board, takes the view that any concession would be paid for out of the pockets of the general body of users.

### A CANADIAN HOSPITAL AMALGAMATION.

THE *Hospital World* of Toronto announces the amalgamation of the Montreal General Hospital and the Montreal Western Hospital. The former institution, which was founded just one hundred years ago, contains 450 beds and twenty-five extra cots for emergency cases, and is the largest hospital in Quebec. The Western is a comparatively young institution, having been founded in 1874; it contains one hundred beds, of which ninety-two are on an average constantly occupied. When the amalgamation is complete the General will be occupied only by public patients, while the Western, which has the necessary spare land, will provide a private patients' pavilion of from 250 to 300 rooms, to be open to any recognised physician or surgeon of good standing in the community who meets the minimum standard requirements of the American College of Surgeons. At the Western will be a large out-patient department, and emergency wards for male and female patients.

### FATAL ACCIDENT AT A HOSPITAL.

A SERIOUS and unusual accident, resulting in the death of an electrician at King's College Hospital, Denmark Hill, occurred last week. A petrol tank belonging to a motor ambulance had been found to be leaking and was dismantled and taken to the hospital's works department for repair. Apparently every precaution was taken and the tank was allowed to remain draining for six hours, after which a fitter commenced to solder the defective part, and almost at once there was an explosion. Curiously enough the fitter himself suffered nothing further than a contused arm and face and was able to proceed home after his injuries had been dressed. The fatal accident occurred to an electrician, who was passing through the shop at the time, and was struck in the neck by a portion of the petrol tank, which penetrated the main artery. Several surgeons were on the spot within a few minutes and the utmost efforts were made to save the man's life, but he died within an hour. An inquest is being held as we go to press.

### "A STANDARD FOR THE WHOLE COUNTRY."

A CONFERENCE expected to have far-reaching effects on health administration and organisation in Wales took place at Swansea on Friday, March 11. It was a meeting attended by thirty delegates from all parts of the Principality of the Welsh Consultative Council of the Ministry of Health, and the principal business was to frame the Council's report to the Ministry on the proposed organisation of the medical and allied services in Wales. The proceedings were private, but it is understood that the Council took into consideration the whole of the services—hospitals, school clinics, mental defectives institutions—and that the immediate upshot of their report will probably be the formation of a separate Welsh area. At a dinner to which the delegates were later entertained by the Mayor of Swansea, the Chairman of the conference, Dr. Ewen MacLean, of Cardiff, gave a hint of the importance of the movement on foot by remarking that the Council in the recommendations they had made were truly sowing the seeds of great developments in Wales in the matter of health. They were absolutely one, he said, in their objective, which was to establish in the Principality a health machine which would set a standard to the whole country.

### THE CASE AGAINST CO-OPERATION.

THE Chairman of the Billericay Guardians, Essex, is reported to have protested against the use of the infirmary by such patients as domestic servants admitted on a medical practitioner's recommendation. The reason alleged is that such cases should be treated in general hospitals, and not at the cost of the rates, and that the practice is an insidious attempt of Dr. Addison to secure the infirmary as a general hospital by subterfuge after having failed to gain it in the open. If such cases are received, the Chairman complains that the infirmary will have to be enlarged, the staff increased, and money spent; in fact, he would rather resign, it appears, than be dictated to by the Minister of Health. The Guardians are deliberating on the matter. We have called this "The Case against Co-operation" because it is the first instance we have noted of active hostility to a plan; and this hostility is interesting, if only because it is the single and invidious exception. Guardians have so long boasted that "destitution" is only a technicality that it is amusing to hear them complain of that which has been their peculiar pride. If the general hospitals were not overcrowded and with long waiting-lists the need for co-operation would not have arisen.

### COALFIELD COTTAGE HOSPITALS.

THE contract for the conversion into a cottage hospital of the mansion at Caerphilly, which was purchased about a year ago by the miners of East Glamorgan has been let to Mr. Robert Moon, a Newport builder, who will carry out the alterations under the supervision of Messrs. Griggs and Vaughan, architects, of the same town. When the conversion is completed and the institution equipped for the treatment of colliery accident cases the miners of East Glamorgan hope to boast of a cottage hospital equal to any in the Welsh or any other coalfield. For some time the men themselves have been levying sixpence a week for the hospital, and this will continue for eighteen months, by which time a sum of £30,000 will have been raised. The colliery owners of the district have supported the project also; £2,750 in all has been subscribed by them.

